# Supplementary figures and images for: Bio-activity prediction of drug candidate compounds targeting SARS-Cov-2 using machine learning approaches
Source: PLoS One. 2023 Sep 5;18(9):e0288053. doi: 10.1371/journal.pone.0288053 (PMC10479925; doi:10.1371/journal.pone.0288053)

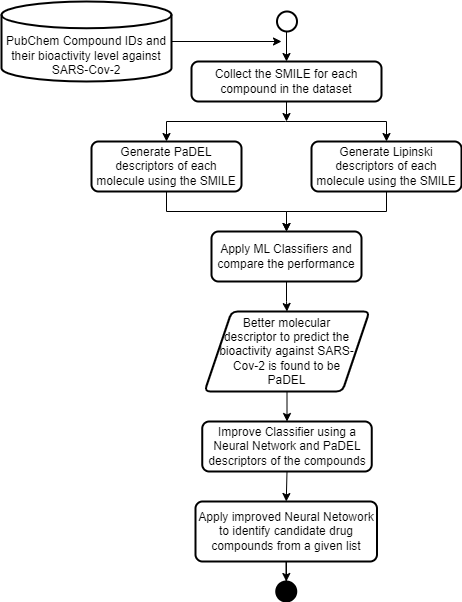

Supplement: S1 Graphical abstract — (PNG) [file pone.0288053.s001.png]
