# Supplementary material for: Bio-activity prediction of drug candidate compounds targeting SARS-Cov-2 using machine learning approaches
Source: PLoS One. 2023 Sep 5;18(9):e0288053. doi: 10.1371/journal.pone.0288053 (PMC10479925; doi:10.1371/journal.pone.0288053)
Supplement: Attachment — Submitted filename: Response to Reviewers.docx [file pone.0288053.s002.docx]

**Reviewer 1**

**Concern 1:**

The authors should provide adequate rational for adopting Lipinski versus PaDEL as comparative tools for calculating molecular descriptive. The comparison isn’t equally footed since, unlike PaDEL, Lipinski’s tool provides only physiochemical parameters and constitutional descriptors, reflecting the ligand’s chemical composition without any information about the molecular geometry nor the atom connectivity. Other molecular descriptor calculators should be used instead like Mordred or else.
Additionally, natural products are often cited as an exception to Lipinski's rules, the thing that would ignore potential candidates.

**Author Response:** We thank the reviewer for the valuable comments. We acknowledge the need for adequacy on the rationalization of our approach.

**Author Action:** We updated the manuscript by adding the following lines in our manuscript:

*“It is crucial to carefully evaluate the selection of descriptor in order to create a representation that works for a deep learning framework. The physical and chemical characteristics of a molecule, such as its solubility, lipophilicity, and molecular weight, are described by physiochemical descriptors. For the design and improvement of drugs, these characteristics may be crucial. On the other hand, molecular descriptors provide a more thorough description of a molecule's structure by taking into account its atom connections and molecular geometry. It is crucial to thoroughly consider the advantages and disadvantages of each approach before selecting the one that is best suited for what we are trying to accomplish”*

**Concern 2:** Authors should provide reference for the criteria used in categorizing the compound’s activity (Active, Inactive, and moderate).

**Author Response:** We thank the reviewer for the valuable comments. We acknowledge the need for the rationale used for categorizing the compound’s activity.

**Author Action:** We updated the manuscript by adding the following lines in our manuscript:

*“It is essential to keep in mind that the IC50 values reported in various research can change according on the experimental setup. For instance, several studies have documented the IC50 values for
the FDA-approved medication remdesivir, which is effective against SARS-CoV-2. Remdesivir had an IC50 of 0.77 μM in Vero E6 cells infected with SARS-CoV-2 [30]. Remdesivir’s IC50 was found to be 0.16 μM in another investigation [31] in human airway epithelial cells infected with SARS-CoV-2. Therefore, the values for this candidate is in the range of 0.16-0.77 μM”*

Further characterization is done in subsequent lines:

*“Based on the IC50 values against SARS-CoV-3CLpro protein, we categorized the data in our curated dataset as active, intermediate, or inactive using the following criteria - Active : IC50 value < 1000nM ,
Intermediate : 1000 ≤ IC50 value < 10, 000nM , Inactive : IC50 value > 10, 000nM . Since powerful inhibitors had IC50 values less than 1000 nM, these threshold values were chosen since models created using them outperformed those discovered.”*

**Concern 3:**  Parameter tuning is generally crucial for machine learning performances. Authors should clearly illustrate their tuning approach

**Author Response:** We thank the reviewer for the valuable comments. We acknowledge the need for the rationale to illustrate the parameter tuning

**Author Action:** We updated the manuscript by adding the following lines in our manuscript:

*“Researchers can use hyperparameter tweaking to fine-tune model performance for the best outcomes. This is a fundamental aspect of machine learning, and selecting optimal hyperparameter values is critical for success. We investigated and experimented with many parameters, including the number of layers, the dropout ratio, and the number of nodes in each layer, and eventually developed an optimum design.”*

**Concern 4:**  Figures are provided without any legend description.

**Author Response:** We thank the reviewer for the valuable comments. We acknowledge the need for legend description.

**Author Action:** We updated the manuscript by adding the legend description to the best of our ability. Even after that, since to err is human, please refer to the exact figure that we might have missed which we will update at reviewers’ discretion.

**Concern 5:**   Author should be careful when converting reference styles to match the submitted journal guidelines. Several paragraphs started by reference numbers rather than the author name. for example, lines 92 and 105. Authors are advised to adequately provide proper in-text citation throughout the whole manuscript.

**Author Response:** We thank the reviewer for the valuable comments. We acknowledge the need for author referencing.

**Author Action:** We updated the manuscript by adding the author names before the citations.

**Reviewer 2**

**Concern 1:** The manuscript entitled “Bio-activity Prediction of Drug Candidate Compounds Targeting SARS-Cov-2 using Machine Learning Approaches” reports evaluation of 27 machine learning classifiers. Authors have developed a neural network model that could identify bioactive and inactive chemicals. Abstract section should also provide a quick overview for the obtained results and its implications. Other than that, study methodologies, results and discussion are well presented.

**Author Response:** We thank the reviewer for the valuable comments. We acknowledge the need for updating the abstract.

**Author Action:** We updated the manuscript by adding the following lines in abstract

*“We also developed a neural network model that can correctly identify bioactive and inactive chemicals with 91% accuracy, on CheMBL data and 93% accuracy on combined data on both CheMBL and Pubchem. The F1-score for inactive and active compounds was 93% and 94%, respectively. SHAP (SHapley Additive exPlanations) on XGB classifier to find important fingerprints from the PaDEL descriptors for this task. The results indicated that the PaDEL descriptors were effective in predicting bioactivity, the proposed neural network design was efficient, and the Explanatory factor through SHAP correctly identified the important fingertips”*

**Reviewer 3**

**Concern 1:** my only comment is that the Figures need to be of higher quality.

**Author Response:** We thank the reviewer for the valuable comments. We acknowledge the need for higher quality figure.

**Author Action:** We updated the manuscript by adding higher quality images. Even after that, since to err is human, please refer to the exact figure that we might have missed which we will update at reviewers’ discretion.
