# Supplementary material for: Bio-activity prediction of drug candidate compounds targeting SARS-Cov-2 using machine learning approaches
Source: PLoS One. 2023 Sep 5;18(9):e0288053. doi: 10.1371/journal.pone.0288053 (PMC10479925; doi:10.1371/journal.pone.0288053)
Supplement: Attachment — Submitted filename: Response to Reviewers.docx [file pone.0288053.s003.docx]

**PONE-D-23-05165R2**

Bio-activity Prediction of Drug Candidate Compounds Targeting SARS-Cov-2 using Machine Learning Approaches

PLOS ONE

Reviewer #1: The authors kindly responded to all raised concerns and recommendations. The can be accepted for publication.

Response: We thank the reviewer for their observations.

Action: No action is taken

**Reviewer #4:** In this study, the authors developed a method to predict the bioactivity of compounds against the SARS-CoV-2 3CLpro protein using machine learning techniques. They utilized PaDEL descriptors and a specially designed neural network model, achieving an impressive 93% accuracy rate. The authors also identified important molecular substructures impacting bioactivity. The results, while promising, require further validation through in-vitro and in-vivo experiments. The manuscript has been written regular with a good discussion.

Thus, this manuscript can be consider to be accepted after minor modification. There are some revisions for better understanding as below:

1) The authors' choice to employ the PaDEL software for generating molecular descriptors is of particular interest. Could the authors elucidate the considerations and comparisons that led them to choose PaDEL, especially when considering potential alternatives such as Gaussian, Dragon, and ChemOffice…? What advantages did PaDEL offer in this particular context that might have superseded the benefits of the aforementioned alternatives?

2) In the Lipinski Descriptors section, please add these references to enrich this part (<https://doi.org/10.1371/journal.pone.0284539>; <https://doi.org/10.1080/07391102.2023.2212304>; )

3) The authors indicate that machine learning techniques have been applied to predict the bioactivity of existing drugs. Could they expand on this point, and possibly discuss any significant findings or implications in this area?

**Response**: We thank the reviewer for their comments. We have summarized our response as 1, 2 and 3 chronologically as per the questions/queries/comments raised by the reviewer.

1. We acknowledge the reviewers concern on addressing why PaDEL is used. To further clarify the stance, following lines were added in the text: ***“Among the software tools commonly used for molecular description and computational chemistry, PaDEL stands out as an excellent choice for high-throughput QSAR modeling. PaDEL's focus on efficient generation of a wide range of molecular descriptors, including 1D, 2D, and 3D descriptors, makes it particularly advantageous for our experimentation. Its ability to swiftly calculate descriptors for large chemical databases enables rapid screening and analysis, while its user-friendly interface ensures accessibility for researchers of varying expertise. With PaDEL, we can efficiently explore the intricate relationships between molecular structures and activities, advancing our understanding of drug design and discovery.”***
2. We acknowledge the reviewers concern to enrich the literature sections. We added the reference within the text. The following lines were added. ***“Baassi and colleagues discusses the in-silico design of a potential new HIV-1 protease inhibitor[13] where the authors reiterated the use of in-silico methods for drug design, which can potentially speed up the drug development process”***
3. We acknowledge the need to expand on pointers on machine learning. We added the following texts to further clarify.

***“Machine learning techniques have emerged as powerful tools for predicting the bioactivity of existing drugs based on their molecular structure and properties. This methodology facilitates the identification of novel drug candidates, repurposing of existing drugs, and optimization of drug design. Different molecular representations, such as fingerprints, descriptors, graphs, and SMILES strings, capture the structural features essential for analysis. These features are then utilized as inputs for diverse machine learning models, including deep neural networks, support vector machines, random forests, and decision trees, which effectively learn the intricate relationship between drug structure and activity. Several studies have reported significant findings in this area, using machine learning techniques to predict the bioactivity of existing drugs. Zhang et al. developed an interpretable machine learning approach to identify novel antibiotics with diverse mechanisms of action, leading to the discovery of bioactive molecules with potential antibacterial activity and novel binding modes. These studies highlight the efficacy of machine learning in predicting bioactivity and its potential in drug discovery. “***
